# Flexible and Wearable PDMS-Based Triboelectric Nanogenerator for Self-Powered Tactile Sensing

**DOI:** 10.3390/nano9091304

**Published:** 2019-09-12

**Authors:** Jie Wang, Shuo Qian, Junbin Yu, Qiang Zhang, Zhongyun Yuan, Shengbo Sang, Xiaohong Zhou, Lining Sun

**Affiliations:** 1 College of Information and Computer, Taiyuan University of Technology, Taiyuan 030024, China; 2 Research Institute of Advanced Manufacturing Technology, Soochow University, Suzhou 215006, China; 3 School of Instrument and Electronics, North University of China, Taiyuan 030051, China; 4 School of Optoelectronic Science and Engineering, Soochow University, Suzhou 215006, China

**Keywords:** triboelectric nanogenerator, tactile sensor, motion detecting, self-powered, ultra-flexible

## Abstract

Flexible electronics devices with tactile perception can sense the mechanical property data of the environment and the human body, and they present a huge potential in the human health system. In particular, the introduction of ultra-flexible and self-powered characteristics to tactile sensors can effectively reduce the problems caused by rigid batteries. Herein, we report a triboelectric nanogenerator (TENG), mainly consisting of an ultra-flexible polydimethylsiloxane (PDMS) film with micro-pyramid-structure and sputtered aluminum electrodes, which achieves highly conformal contact with skin and the self-powered detection of human body motions. The flexible polyethylene terephthalate (PET) film was selected as spacer layer, which made the sensor work in the contact-separation mode and endowed the perfect coupling of triboelectrification and electrostatic induction. Moreover, the controllable and uniform micro-structure PDMS film was fabricated by using the micro-electro-mechanical system (MEMS) manufacturing process, bringing a good sensitivity and high output performance to the device. The developed TENG can directly convert mechanical energy into electric energy and light up 110 green Light-Emitting Diodes (LEDs). Furthermore, the TENG-based sensor displays good sensitivity (2.54 V/kPa), excellent linearity (*R*^2^ = 0.99522) and good stability (over 30,000 cycles). By virtue of the compact size, great electrical properties, and great mechanical properties, the developed sensor can be conformally attached to human skin to monitor joint movements, presenting a promising application in wearable tactile devices. We believe that the ultra-flexible and self-powered tactile TENG-based sensor could have tremendous application in wearable electrons.

## 1. Introduction

As an important part of wearable devices and medical care devices, flexible electronics have drawn great attention in various fields, including tactile sensors [1], energy supply devices [2,3] and flexible displays [4]. In particular, tactile sensors have had widespread interest due to their ability to mimic human skin. Based on the principle of force–electricity conversion, the flexible tactile sensors can be divided into piezoelectric, capacitive, piezoelectric, and triboelectric types. In the past ten years, many strategies have been reported to realize high sensitivity, good linearity and good flexibility. In particular, some studies have developed several micro-structures (silk-based [5], leaf-based [6], interconnected networked [7,8], bionic structured [9] and double arched [10]) to improve device sensitivity and output performance. Nevertheless, the energy supply is still an integral component of these devices. The traditional rigid battery has resisted the applications of flexible tactile devices because of its un-comfort, low durability and low service time [11,12]. Hence, there is a pressing need in the field of wearable devices to develop self-powered sensing devices.

Self-powered devices could directly convert mechanical energy into electrical responses from ambient mechanical energy, which is a promising solution for the next-generation flexible wearable devices. With the rapid development of nanoscience and nanotechnology, various types of nanogenerators (piezoelectric-based, triboelectric-based, thermoelectric-based and magnetoelectric-based) have been reported [2,3,13]. Among them, the piezoelectric nanogenerator (PENG) and the triboelectric nanogenerator (TENG) present great potential both in energy harvesting and mechanical sensing. Most piezoelectric materials are generally not inherently flexible [14,15]. The common solution for bring flexibility to piezoelectric materials is to reduce its thickness through chemical mechanical polishing (CMP) technology [16,17] and to prepare the thin films on rigid substrate by the sol-gel method [1,18]. However, such a PENG cannot satisfy the demand of mechanical sensing due to its poor performance under a large deformation. Another way is to prepare a piezoelectric composite film by integrating rigid piezoelectric materials into a flexible polymer that can realize both piezoelectricity and flexibility [19,20]. Though this strategy realizes the flexibility of piezoelectric materials, the preparation process is still complicated. Due to the advantages of easy fabrication and not needing pre-treatment, the triboelectric nanogenerator (TENG), which is coupled of the triboelectrification and electrostatic induction, presents great potential in energy harvesting and mechanical sensing [3]. Since Wang et al. [21] first invented the TENG in 2012, it has been used in various power supply and wearable sensors in recent years. However, there are still some challenges in developing TENG-based flexible electronics. Hou et al. [3] reported on a stretchable triboelectric textile for harvesting multivariant human motion energy. Though this novel device presents great electrical output performance and potential applications in monitoring human joint movement, it shows poor conformal contact with human skin and wearable clothes. Chen et al. [22] demonstrated a single-electrode triboelectric-nanogenerator for sensing instantaneous force, yet the single-electrode mode was easily disturbed by the surrounding environment. Hence, combining flexible materials with the contact-separation mode TENG to monitor human body movement and harvest human motion energy is an urgent direction for developing self-powered tactile sensing devices.

Polydimethylsiloxane (PDMS) is considered to be an ideal material for flexible wearable electronics due to its inherent elasticity and excellent biocompatibility [5]. PDMS can be discretionarily twisted, compressed, deformed and stretched, and it has drawn increasing attention in materials science and mechanical engineering. By introducing a micro-structure (leaf-based [9], spherical [23] and so on) and integrating various sensitive materials (graphene [24], Carbon nanotube (CNT) [25], metal nanowires [26] and so on) on the PDMS surface, the sensors with PDMS present excellent sensitivity, good linearity and perfect flexibility. Moreover, the micro-structure of the PDMS membrane can effectively reduce the problem of visco-elastic behavior and thus increase its friction performance. What is more, the micro-electro-mechanical system (MEMS) manufacturing process is a promising technology to fabricate an Si mold for preparing the PDMS membrane with a controllable and uniform micro-structure [27]. Therefore, for the TENG, in order to combine excellent flexibility and high output performance, it is urgent to use the PDMS elastomer as the main body and improve the specific surface area of the friction layer by MEMS technology.

Here, we developed a self-powered flexible and wearable sensor based on the contact-separation TENG, one which could fully contact human skin and be used for harvesting mechanical energy and detecting human motion. Through the introduction of a spacer layer, the triboelectrification and electrostatic induction could work simultaneously. The elastic PDMS material simultaneously acted as the main body and the one triboelectric layer, endowing the sensor with great flexibility. The controllable and uniform micro-structure was fabricated by the MEMS manufacturing technology. By relying on the micro-structure on the friction layer surface, the sensor displays good sensitivity (2.54 V/kPa), excellent linearity (*R*^2^ = 0.99522) and good stability (over 30,000 cycles). Due to its compact size, great electrical properties, and great mechanical properties, the developed sensor can be conformally attached to human skin to monitor joint movements, presenting a promising application in wearable tactile devices. Overall, the proposed flexible TENG-based sensor is favorable for harvesting human mechanical energy and detecting human motion, which may broaden their potential applications in energy harvesting and self-powered sensing.

## 2. Experimental Section

### 2.1. Material and Device

Materials: A buffered oxide etch solution (BOE, NH_4_F:HF = 6:1), acetone (AR), tetramethylammonium hydroxide (TMAH, AR), PDMS elastomer (Sylgard 184 was purchased from Dow Corning Inc., Midland, MI, USA), a 4 inch (100) Si wafer, the RZJ-304 photoresist, and the developing solution were purchased from ALADDIN Inc., Shanghai, China.

**Figure 1:**
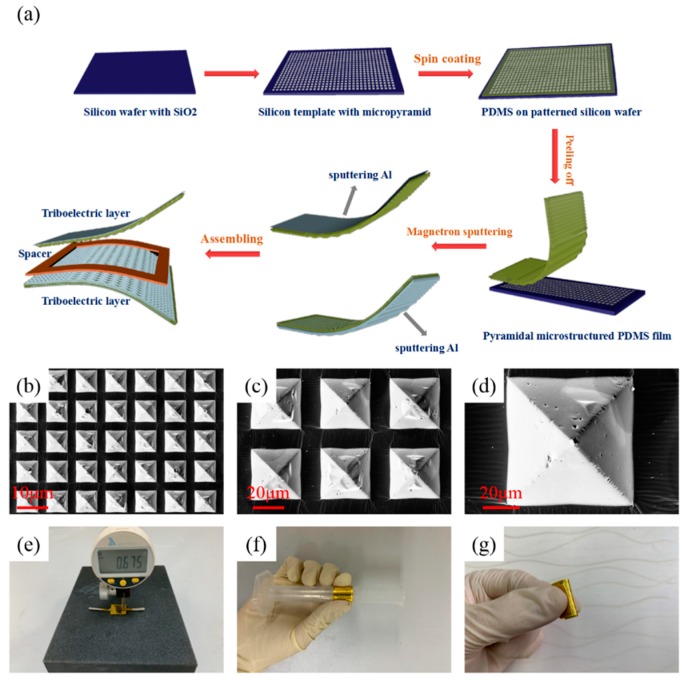
(**a**) The fabrication process of the triboelectric nanogenerator (TENG)-based sensor. (**b**–**d**) The SEM images at different scales. (**e**) Photograph of the TENG-based sensor, which shows its thickness (~0.7 mm). (**f**,**g**) Photographs of the TENG-based sensor, which shows its excellent flexibility.

Preparation of Micro-Pyramid-Structure PDMS Films*:* As shown in Figure 1a, the patterned Si wafer was fabricated by lithography. Then, the exposed SiO_2_ patterns were etched by the BOE solution. Next, the micro-pyramid-structure was fabricated by using the anisotropic etching at 90 °C for 50 min, and, then, the remaining SiO_2_ layer was etched by the BOE solution to obtain the Si mold. The PDMS base was mixed with the curing agent (10:1), and the mixture was degassed in vacuum for 30 min to remove bubbles. Then, the mixture was spin-coated onto the Si mold and followed by solidifying at 90 °C for 30 min. Finally, the PDMS film was peeled off from the Si mold.

Fabrication of TENG-Based Self-Powered Sensor: The 200 nm Al was sputtered on the front of the micro-pyramid-structure PDMS film as both the friction layer and electrode, and the 200 nm Al was sputtered on the back of another micro-pyramid-structure PDMS film as another electrode. Then, a spacer layer (polyethylene terephthalate—PET) was placed between the two PDMS films. Next, the conductive fabric was placed on the Al film to make an external contact. Finally, the device was covered by the pure PDMS film, which acted as the protective layer.

### 2.2. Characterization and Measurement

The sample morphologies were characterized by a field emission scanning electron microscopy (ZEISS EVO18, Carl Zeiss Jena, Germany). The optical images were captured using a Sony Camera. The output voltage and current were measured with the Source Meter 2611B test system (Keithley Instruments, Inc., Cleveland, OH, USA).

## 3. Results and Discussion

Figure 1a demonstrates the main fabrication process of the flexible TENG-based sensor. First, the pattern of mask plate was transferred to the Si wafer (with SiO_2_ layer) by lithography. Then, the wafer was wet etched by the BOE solution (buffered oxide etch, NH_4_F:HF = 6:1, *v*/*v*), and the photoresist was removed by the acetone. Next, the wafer was wet etched using the TMAH solution at 90 °C for 60 min. Then, the remaining SiO_2_ layer was removed by the BOE solution to obtain the Si wafer with the micro-pyramid-structure. Next, the PDMS elastomer and the curing agent were mixed at a weight ratio of 10:1 and stirred for 20 min, and then they were spin-coated on the Si wafer (800 rpm for 20 s). After curing at 90 °C for 30 min, the PDMS film was peeled off from the Si wafer. Then, the Al film (200 nm) was sputtered on the back and front of two PDMS films. Finally, the spacer layer (PET, 50 μm) was placed at the middle of two PDMS films, and the device was coated by the plane PDMS film. In this work, as shown in Table S1, PDMS acted as the negative friction material due to its great ability to gain electrons, and Al acted as the positive friction material due to its great ability to lose electrons. Overall, the developed sensor consisted of two triboelectric layers with electrodes and a spacer layer. Figure 1b–d show the scanning electron microscopy (SEM) images of the PDMS film with the micro-pyramid-structure (70 × 70 × 42 μm at intervals of 27 μm) at different magnifications. The uniform and high-density topography of the PDMS surface greatly affect the output of the sensor. Digital photographs of this TENG-based sensor are shown in Figure 1e, and these indicate that it has a thin thickness. Figure 1f,g show the sensor was wrapped around a glass rod and bent by hand, which indicate that it has excellent flexibility. The small size and light weight of the sensor are shown in Figure S1a–c.

The working mechanism of the developed TENG-based sensor is the same as that of other contact-separation mode TENGs [28,29]. As shown in Figure S2, when the two friction layers contact and rub with each other, the equal amount of charges with opposites polarities accumulate on the surface of two friction layers. The positive charges will accumulate on the Cu surface, and negative charges will accumulate on the PDMS surface due to the triboelectric effect. When the two friction layers are separated from each other, the electrons flow from the PDMS film to the Cu film through an external circuit to balance the static charges. The alternating signals are generated by way of the continuous contact-separation of the two friction layers. If we define the relative dielectric constant of the cavity as *ε_c_*, the surface charge density is *σ*, and the distance of the two triboelectric layers is *x*(*t*). Then, the open-circuit voltage *V*_OC_ can be expressed as:

$$V_{OC}=\frac{\sigma x\left(t\right)}{\varepsilon_{c}}$$

Hence, the output voltage is directly related to the surface charge density and the distance of the two triboelectric layers. The introduction of a micro-pyramid-structure on the friction layer surface is an effective way to increase the specific surface area and thus enhance the surface charge density during the contact of the two friction layers [30]. What is more, aluminum and PDMS were selected as the triboelectric pair because they are located at the opposite ends of the triboelectric series. Hence, they show an excellent capacity to lose or gain electrons when contacted with each other.

**Figure 2:**
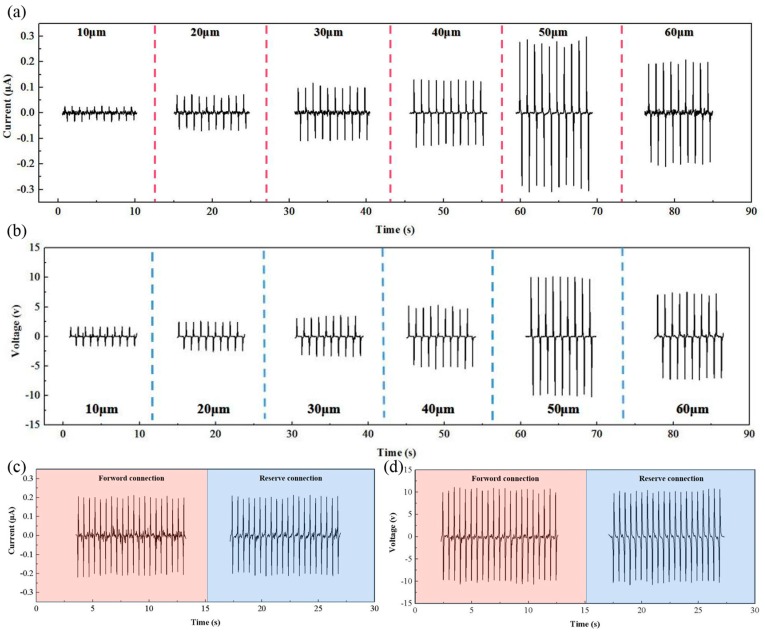
(**a**,**b**) The short-circuit current (*I*_SC_) and open-circuit voltage (*V*_OC_) of the sensor under the different spacer thicknesses, respectively (10, 20, 30, 40, 50 and 60 μm). (**c**,**d**) The *I*_SC_ and *V*_OC_ of the sensor with forward/reverse connection modes.

According to the above equation, the distance of two friction layers (spacer thickness) also affects the sensor’s output performance. To characterize the output of the sensor under different spacer thicknesses, a linear motor was used to provide quantitatively periodic external forces to apply to the sensor. The measurement (2611B) was used to precisely obtain the output voltage and output current. The sensor size was 2.0 × 1.8 cm^2^; the effective contact area was 1 × 1 cm^2^; and the applied periodic force was 10 N during the electrical test. Figure 2a,b respectively, present the short-circuit current (*I*_SC_) and the open-circuit voltage (*V*_OC_) performances of the sensor under different spacer thicknesses. Both *I*_SC_ or *V*_OC_ showed the tendency of first increasing and then decreasing, and the maximum output could be obtained under the 50 μm spacer thickness. However, according to the above formula, the output was proportional to the spacer thickness. The test results did not match the theory. This difference was due to the fact that sensor could not be equivalent to the parallel plate capacitor with the increase of spacer thickness [31]. That is to say that the edge effect of capacitance directly affected the sensor output performance. Hence, the thickness of spacer layer was selected as 50 μm to obtain the optimal output performance. Figure 2c,d present the *I*_SC_ and *V*_OC_ of the sensor with different connection modes (forward connection and reserve connection), which demonstrate that the output electric signals truly belonged to the device.

**Figure 3:**
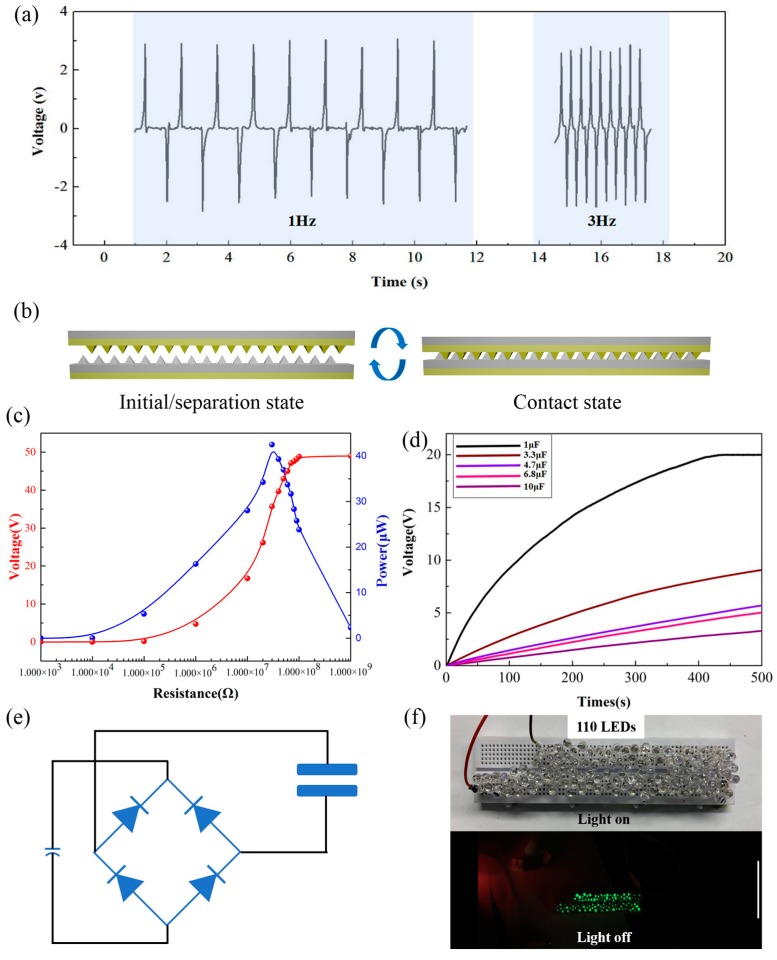
(**a**) The open-circuit voltage of the developed TENG at different motion frequencies (1–3 Hz). (**b**) The schematic diagram of the contact-separation mode under the charging curves test and the lighting test. (**c**) The variation curve of the pear power and output voltage with the different external loads (the effective contact area was 4 × 4 cm^2^). (**d**) The charging curves of capacitors charged by the developed TENG (the effective contact area was 4 × 4 cm^2^). (**e**) The schematic of bridge rectifier circuit. (**f**) The 110 LEDs were directly lit up by the developed TENG under the contact-separation process.

Considering its practical application, the sensor is supposed to be operated under different frequencies. Figure 3a presents the electrical output performances of the sensor at the operating frequencies from 1 to 3 Hz. The results show that the peak-to-peak value of the *V*_OC_ was independent of the frequency. According to the previous research, the influencing factors of *V*_OC_ are the amount of transfer charges and the capacitance between two electrodes at different spacer thicknesses [3,32]. Therefore, the *V*_OC_ is irrelevant to the frequency (time). In order to evaluate the output power, the TENG usually connects with external loads from 1 KΩ to 1 GΩ in series as a power supply (the effective contact area was 4 × 4 cm^2^ in the test; as shown in Figure S3, the two triboelectric layers were separately fastened on the fixed end and active end; the schematic diagram of contact-separation mode is shown in Figure 3b). The output power can be calculated from *V*_OC_^2^/*R*, where *R* is the external load resistance. As shown in Figure 3c, the tendency of output power first increased and then decreased; the power could reach up to maximum when the external load resistance was about 30 MΩ. Capacitors are a common energy storage unit which can store the power energy generated from the developed TENG. The charging curves (Figure 3d) show the time required for the developed TENG to charge different capacitors via a bridge rectifier circuit. The schematic diagram of the bridge rectifier circuit is shown in Figure 3e. The alternating electricity could charge a 1 μF capacitor to ~20 V in seven min with the continuous contact-separation process. The charging time increased as the value of the capacitor increased. Then, the stored energy in capacitor could further support the power for the commercial equipment (calculator, hygrothermograph, electronic watches and so on). Furthermore, as a force–electricity conversion device, we utilized 110 green LEDs that connected in series as an external load to validate the feasibility of the developed TENG as a power source. As shown in Figure 3f and Video S1, the 110 LEDs could be directly lit up when the TENG performed the continuous contact-separation process.

**Figure 4:**
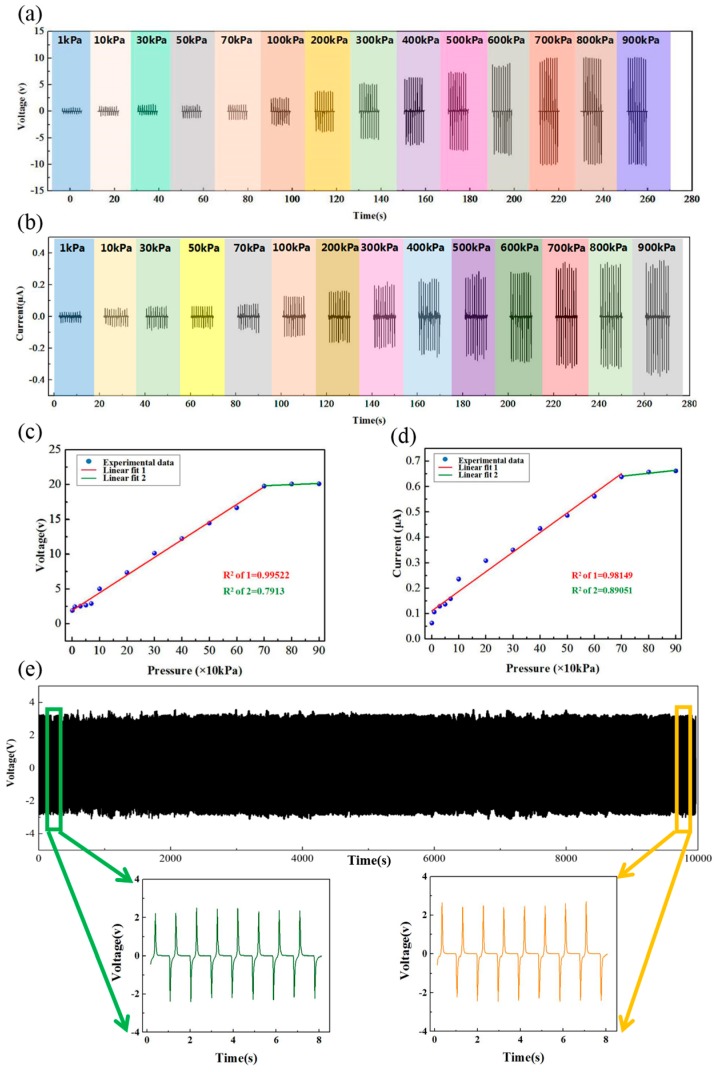
(**a**) The open-circuit voltage of the sensor under different applied forces. (**b**) The short-circuit current of the sensor under different applied forces. (**c**) The linear fitting analysis of peak-to-peak open-circuit voltage. (**d**) The linear fitting analysis of peak-to-peak short-circuit current. (**e**) The stability test under the cycled loading and unloading (insets show the enlarged first and last eight cycles).

Sensitivity, linearity and stability are important indexes to evaluate sensor performance. As shown in Figure 4a,b, we tested the electric performances of the TENG-based sensor under different pressures (1–900 kPa). The relationship between pressure and peak-to-peak voltage/current is shown in Figure 4b,c. After the data fitting process, it is clearly shown that the sensor had good linearity of 0.99522 in the voltage and 0.98149 in the current in the low-pressure region (1–900 kPa). In the high-pressure region, the linearity looked very high, but the fitting data were very low (0.7913 in the voltage and 0.89051 in the current). The reason for this is that there were not enough sufficient data points (samples). Additionally, a stability test under the cycled pressure was conducted and displayed no obvious decline until 30,000 cycles (the pressure: 100 kPa; Figure 4e). The inset of Figure 4e shows the enlarged graphs of start and end cycles. The image of the sensor after the stability test is shown in Figure S4a, and the weight of the sensor is shown in Figure S4b. Figure S4c shows the image of the micro-structure, indicating that the surface micro-structure had no obvious change.

**Figure 5:**
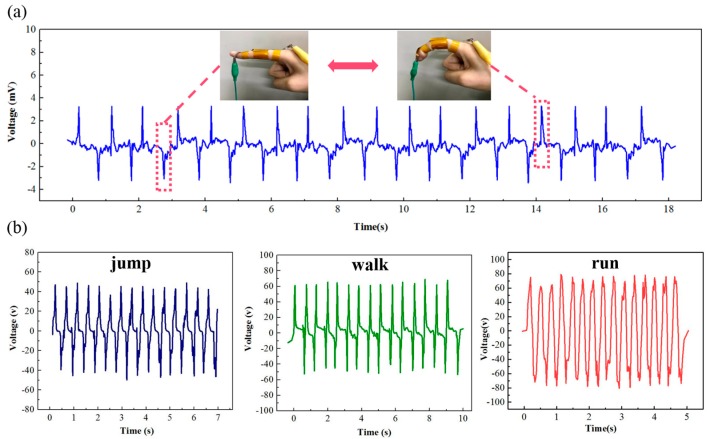
The sensing properties of the developed TENG at different positions of the human body: (**a**) At the knuckle and (**b**) at the knee joint.

Due to its excellent electric performance (high output, high sensitivity and good linearity), high flexibility and good stability, the developed TENG-based sensor can be easily attached to the human joint or cloth, and it can used for various mechanical energy harvests and human body movement detections. As shown in Figure 5a, the TENG-based sensor can be fixed to the finger by 3M tape to monitor bending status in real time. The voltage can reach up to ±4V, and the positive voltage and negative voltage correspond with the bending status and release status, respectively. Furthermore, the sensor can not only monitor finger movement but also detect different motion states. For instance, the developed sensor can be attached on the knee to detect different motion states. The bending of knee brings the contact and separation of the two triboelectric layers; the different motion states lead to the different peak-to-peak values and frequencies of the obtained signals. Hence, as shown in Figure 5b and Videos S2 and S3, the peak-to-peak values of the obtained signals were as follows: Jump < walk < run; the frequencies were follows: Walk < jump < run. Hence, we can obtain the detailed movements information from the shapes and frequencies of the obtained signals. Consequently, the above results demonstrate the promising application of the flexible and self-powered TENG-based sensor in harvesting human mechanical energy and detecting human motion.

## 4. Conclusions

In summary, this paper demonstrated a novel triboelectric nanogenerator composed of two micro-structured PDMS films and a spacer layer. The PDMS elastomer brings the highly skin-conformal characteristics to the developed TENG device. The working principle of the triboelectric effect induced by interaction between micro-structured PDMS and Cu films was fully analyzed. The introducing of the micro-pyramid-structure on the PDMS surface allows for excellent electric output performances. When cycled pressure is applied, the developed TENG can charge the 1 μf capacitor for seven min and directly light up 110 green LEDs with a real contact area of 4 × 4 cm^2^. Further considering its excellent output performance and high flexibility, the developed TENG can be easily attached to the human joints to monitor posture changes in real-time. Due to its wearable and flexible nature, the well-designed TENG provides a new way to achieve a power supply for wearable electronic devices and act as an active sensor to monitor human gesture movements.

## Supplementary Materials

The following are available online at https://www.mdpi.com/2079-4991/9/9/1304/s1. Figure S1: The size and weight of the developed sensor, Figure S2: The schematic diagram of the test system. Table S1: The triboelectric series, Video S1: The sensor can directly light up 100 LEDs, Video S2 and S3: The sensor was attached onto the knee to monitor the motion state.
