# Supplementary material for: Flexible and Wearable PDMS-Based Triboelectric Nanogenerator for Self-Powered Tactile Sensing
Source: Nanomaterials (Basel). 2019 Sep 12;9(9):1304. doi: 10.3390/nano9091304 (PMC6781082; doi:10.3390/nano9091304)
Supplement: Supplementary file 1 [file nanomaterials-09-01304-s001.zip › nanomaterials-581624 supplementary materilas send to XML .pdf]

Flexible and Wearable PDMS-Based Triboelectric Nanogenerator for Self-Powered Tactile Sensing

Jie Wang [1,2,*,†], Shuo Qian [3,†], Junbin Yu [3], Qiang Zhang [1], Zhongyun Yuan [1], Shengbo Sang [1], Xiaohong Zhou [4] and Lining Sun [2]

1 College of Information and Computer, Taiyuan University of Technology, Taiyuan 030024, China
2 Research Institute of Advanced Manufacturing Technology, Soochow University, Suzhou 215006, China
3 School of Instrument and Electronics, North University of China, Taiyuan 030051, China
4 School of Optoelectronic Science and Engineering, Soochow University, Suzhou 215006, China
* Correspondence: wangjie@tyut.edu.cn
† These authors contributed equally to this work.

Received: 10 August 2019; Accepted: 9 September 2019; Published: date

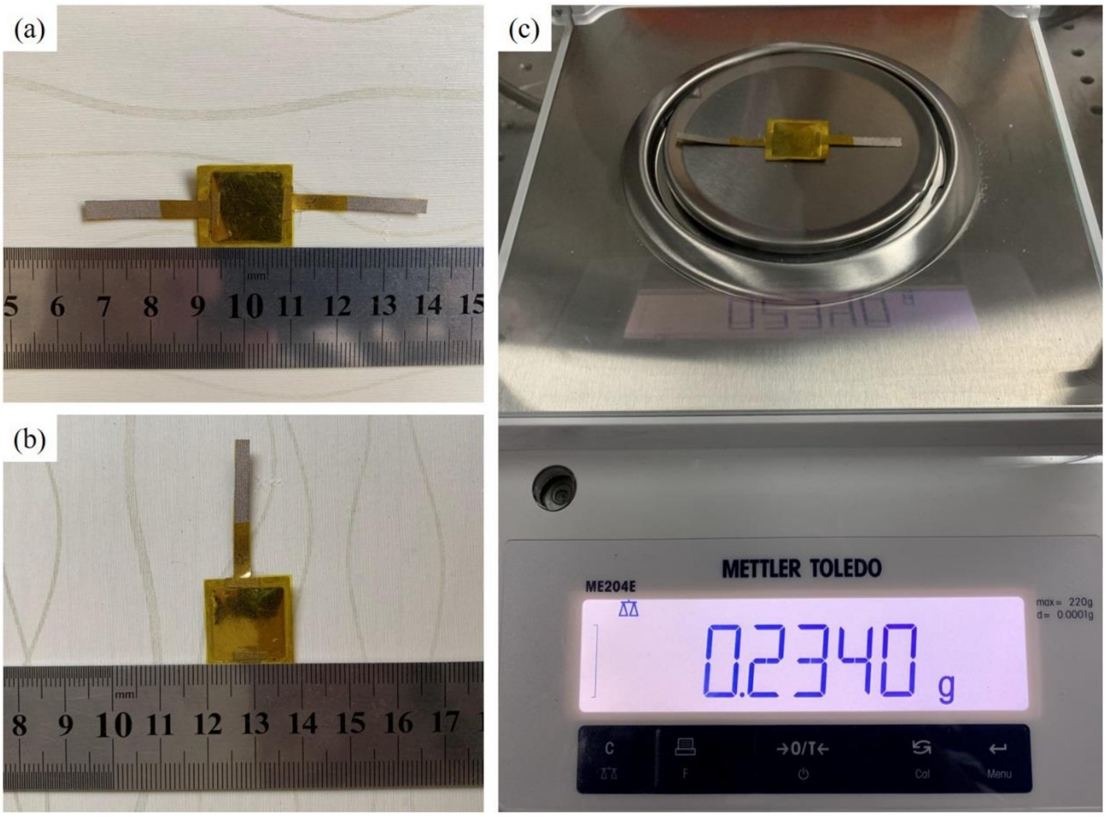

Figure S1. (**a**, **b**) The size of the developed sensor; (**c**) The weight of the developed sensor.

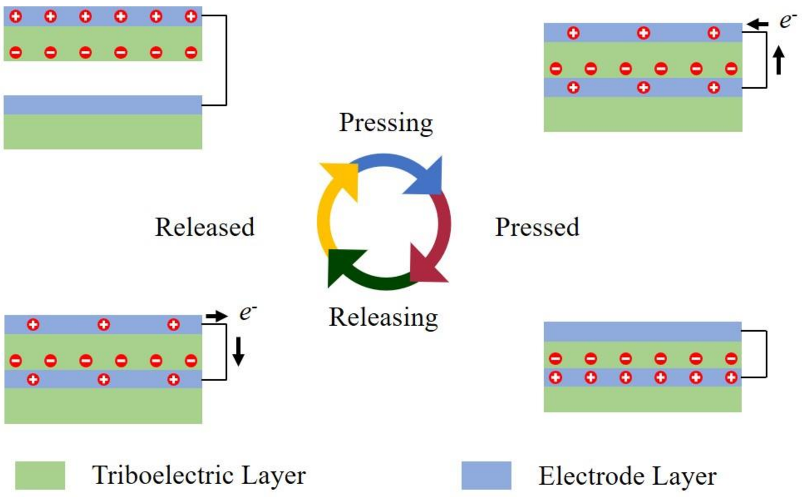

Figure S2. The working mechanism of TENG-base sensor.

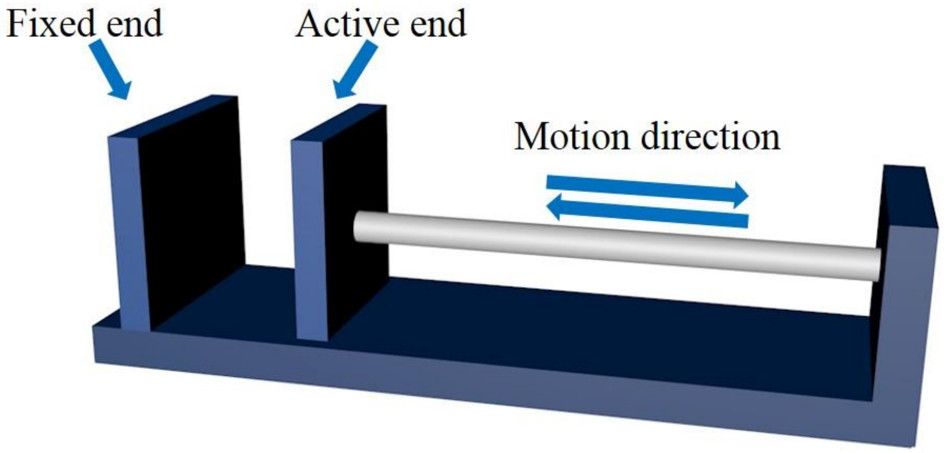

Figure S3. The schematic diagram of the charging curves test and lighting test.

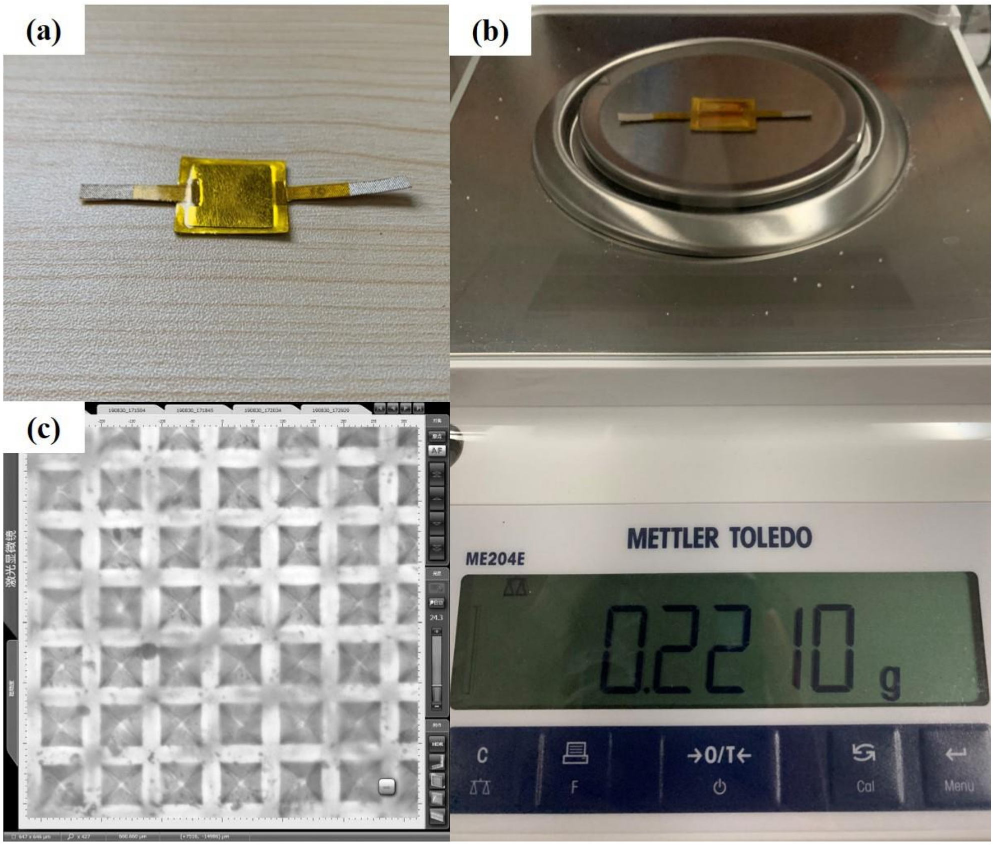

Figure S4. (**a**) The image of the developed sensor; (**b**) The weight of the developed sensor; (**c**) The image of micro-structure after the stability test (tested by the confocal microscopy).

Table S1. The triboelectric series.

**Positive**	**Polyformaldehyde 1.3-1.4**	**（Continued）**	
	Etycellulose	Polyester	
	Polyamide 11	Polyisobutylene	
	Polyamide 66	Polyurethane flexible sponge	
	Melanime formol	Polyethylene terephthalate (PET)	
	Wool, knitted	Polyethyl butyral	
	Silk woven	Polychlorobutadience	
	aluminum	Natural rubber	
	Paper	Polyacrionitrite	
	Cotton, woven	Acrylonitrile vinyl chloride	
	Steel	Polybispheol carbonate	
	Wood	Polychloroether	
	Hard Rubber	Polyvinylidene chioride	
	Nickel, copper, mercury	Polystyrene	
	Sulfur	Polyethylene	
	Brass, silver	Polypropylene	
	Acetate, Rayon	Polyimide (Kapton)	
	Polymethyl Methacrylate (PMMA)	Polyvinvi chloride (PVC)	
	Polyvinyl alcohol	Polyvinvi chloride (PVDF), polydimethylsiloxane (PDMS)	
	(continued)	Polytetrafluoroethylene (PTFE)	**Negative**
